# Research Progress on the Interaction Between SIRT1 and Mitochondrial Biochemistry in the Aging of the Reproductive System

**DOI:** 10.3390/biology14060643

**Published:** 2025-06-02

**Authors:** Yang Li, Kai Kang, Huimingda Bao, Siqi Liu, Bangyi Zhao, Guangdong Hu, Jiang Wu

**Affiliations:** 1 College of Coastal Agricultural Sciences, Guangdong Ocean University, Zhanjiang 524088, China; 2112404111@stu.gdou.edu.cn (Y.L.); kangk@gdou.edu.cn (K.K.);; 2 College of Animal Science and Technology, Shihezi University, Shihezi 832000, China

**Keywords:** interaction, sirtuins, mitochondrial biochemistry, aging, reproductive system

## Abstract

The protein associated with the silencing information regulator 2-associated enzyme1 (SIRT1) is a highly conserved nicotinamide adenine dinucleotide (NAD^+^)-dependent deacetylase and a key member of the sirtuin family. SIRT1 plays an essential role in various cellular physiological processes, primarily localized in the nucleus but also active in the cytoplasm and mitochondria. Recent studies have demonstrated its capacity to delay aging in multiple organs and tissues, although its underlying mechanisms remain incompletely understood. Additionally, SIRT1 exerts a significant influence on metabolic regulation and genetic processes. As the primary source of cellular energy, mitochondria are central to numerous biological functions. Mitochondrial dysfunction has been implicated in the onset and progression of various diseases and is increasingly recognized for its role in aging-related processes. This article investigates the interaction between SIRT1 and mitochondria in regulating reproductive system aging and elucidates their potential mechanisms of action, providing insights for clinical research into reproductive system aging.

## Simple Summary

SIRT1 is one of the most extensively investigated factors in aging research. It plays a pivotal role in various cellular physiological processes and significantly modulates mitochondrial function. As a critical organelle, mitochondria are closely linked to the initiation and progression of multiple diseases through their dysfunction. In recent years, the role of mitochondria in the aging process has increasingly become a central focus of research. Accumulating evidence demonstrates that the interaction between SIRT1 and mitochondria plays an essential role in the aging of organs across different tissues. Consequently, this article aims to explore the relationship between SIRT1, mitochondria, and aging, as well as the mechanisms by which SIRT1 participates in mitochondrial biogenesis, to elucidate its impact on reproductive system aging and provide insights for delaying the aging of the reproductive system.

## 1. Introduction

Aging represents a complex biological process influenced by multifactorial and hierarchical interactions. It encompasses irreversible changes driven by endogenous and environmental factors across all organ and systemic levels. These changes include increased DNA damage, chronic inflammation, and metabolic dysfunction, which collectively contribute to the aging phenotype [1,2]. Aging is currently defined as a progressive decline in the functional capacity of various organs and tissues throughout the lifespan of an organism, occurring universally across different life stages. The aging of the reproductive system constitutes one of the inevitable components of organismal aging. With the intensification of global population aging, research on reproductive system aging is of great significance for addressing challenges in society, the economy, and other areas. For females, reproductive system aging can lead to a decrease in ovarian vitality, reduced egg production, and ultimately a significant decline in or loss of fertility; for males, it can result in decreased sperm production, reduced motility, and increased abnormality rates, ultimately affecting fertility. Multiple factors are believed to contribute to reproductive aging, including oxidative stress, mitochondrial defects, telomere shortening, errors in chromosome separation during meiosis, and genetic alterations [3].

Sirtuin1 (SIRT1) is a highly conserved NAD-dependent deacetylase belonging to the sirtuin family, acting as a post-translational regulatory factor and one of the most active proteins in current aging research [4]. There is also a strong mutual influence between mitochondrial function and aging; mitochondrial dysfunction and cellular senescence are markers of aging [5,6]. Therefore, we searched for relevant academic papers over the past 20 years in databases such as Scopus, SCIE (Web of Science), PubMed, and other databases, using aging, SIRT1, mitochondrial biogenesis, and the reproductive system as our search keywords. This article reviews the research progress on SIRT1 interacting with mitochondria to regulate the aging of the reproductive system, aiming to provide insights for clinical studies on delaying the aging of the reproductive system.

## 2. SIRT1, Mitochondrial Biogenesis, and Senescence

### 2.1. Typing of the Sirtuins Gene

Sirtuins form a highly conserved family of NAD^+^-dependent deacetylases, also known as Class III histone deacetylases. They play a crucial role in regulating various biological processes by removing acetyl groups from lysine residues on proteins, which affects protein activity, stability, and interactions with other molecules. In mammals, there are seven known subtypes of sirtuins, named SIRT1 through SIRT7, each exhibiting significant functional and locational differences [7]. An increasing body of evidence indicates that all seven members of the SIRT family are pivotal in both health maintenance and disease pathology [8]. SIRT1 and SIRT2 are predominantly nuclear but can also migrate between the nucleus and cytoplasm; SIRT3, SIRT4, and SIRT5 are mitochondrial proteins that govern energy metabolism and are found within mitochondria; and SIRT6 and SIRT7 are predominantly nuclear [2,9].

SIRT1 and SIRT2 are pivotal in cell metabolism, inflammation, aging, cardiovascular diseases, neurodegenerative disorders, and cancer. SIRT1, a key factor in maintaining cellular homeostasis and health, has garnered increasing attention over the past two decades [10,11,12,13]. SIRT3, located in the mitochondria, also plays a role in regulating various cellular processes, including energy metabolism, mitochondrial biogenesis, and antioxidant stress. The crirole of SIRT3 in multiple diseases has made it a potential therapeutic target [14]. SIRT4, less studied within the sirtuins family, has been reported to have important physiological functions in organisms, such as promoting DNA damage repair, participating in the energy metabolism of three substances, inhibiting inflammatory responses and apoptosis, and regulating mitochondrial function [15]. SIRT5, another member of the sirtuin family found in mitochondria, has low deacetylation activity and primarily regulates protein function through modifications such as de-oleosylation, de-propionylation, and de-pentadecanoylation, participating in various physiological and pathological processes; the dysregulation or uncontrolled activity of SIRT5 can lead to multiple human diseases, including cancer, Alzheimer’s disease, and Parkinson’s disease, making SIRT5 a promising drug target [16]. Since its discovery, SIRT6 has garnered particular attention. Studies have shown that animals lacking SIRT6 exhibit a series of symptoms including genomic instability, metabolic disorders, and premature death. Therefore, SIRT6 has long been considered a longevity protein [17]. SIRT6 dysregulation is associated with many diseases, including cancer, cardiovascular disease (CVD), neurodegenerative diseases, and diabetes. Currently, SIRT6 has been identified as a key factor in the onset and progression of cancer and CVD [18]. SIRT7 is the only mammalian sirtuin member primarily residing in the nucleolus, and is believed to be involved in ribosome biogenesis, aging, and cellular stress responses. The ablation of SIRT7 will induce overall genomic instability, premature aging, metabolic dysfunction, and reduced stress resistance, thus playing a crucial role in anti-aging processes [19].

SIRT1, the most extensively studied member of the sirtuin family, has been shown to play a role in regulating various important biological processes, such as inflammation, mitochondrial biogenesis, and cellular senescence, which is associated with aging [20]. Comprising 747 amino acid residues, SIRT1 mainly consists of three domains: its conserved catalytic core, a C-terminal domain, and a N-terminal domain. The catalytic core, which is the central functional region of SIRT1, spans amino acid residues 244–412; the COOH-terminal and NH2-terminal regions are made up of residues 1–180 and 513–747, respectively [21]. Furthermore, SIRT1 contains a nuclear localization signal (KRKKRK) [22] within residues 41–46, explaining why SIRT1 is classified as a nuclear protein. Despite its nuclear location, SIRT1 can also regulate target proteins in the cytoplasm. It primarily acts as an acetyltransferase rather than an acetylhydrolase, specifically cleaving the nicotinamide ribosyl bond of NAD^+^ and transferring the acetyl group from proteins to their co-substrates via ADP-ribose-peptidylisomerase intermediates [23]. According to current research, SIRT1 can produce various transcripts and protein variants through alternative splicing, and its role is increasingly recognized. For instance, SIRT1-ΔExon8 is an alternative splicing variant of SIRT1 that lacks its eighth exon. Research has indicated that SIRT1-ΔExon8 is widely expressed in the tissues of both healthy individuals and mice. Compared to the classic full-length SIRT1 (SIRT1-FL), the SIRT1-ΔExon8 variant maintains basic deacetylase activity and exhibits unique stress responsiveness, RNA/protein stability, and protein–protein interactions. Additionally, SIRT1-ΔExon8 can regulate p53 activity, which in turn influences SIRT1 splicing variants [24].

### 2.2. The Relationship Between SIRT1 and Aging

Aging is an extremely complex biological process regulated by multiple factors. Its mechanisms are intricate and involve a wide range of levels. The characteristic of aging is the gradual loss of tissue and organ function due to genetic and environmental factors, as well as nutrition and lifestyle [25]. In recent years, theories about aging have emerged one after another, primarily including the free radical theory, telomere shortening theory, gene regulation theory, and DNA damage theory. Among these, SIRT1 from the sirtuin family is one of the most actively studied regulatory factors in aging research, with various anti-aging effects.

Recent studies have indicated that a deficiency in SIRT1 results in heightened levels of oxidative stress, leading to the production of significant amounts of reactive oxygen species (ROS). This, in turn, causes cellular and organ damage and accelerates the aging process. The human antioxidant system is regulated by various signaling pathways, including the AMPK-SIRT1-FOXO pathway. Under conditions of oxidative stress, SIRT1 can counteract the forkhead box protein O (FOXO) family of transcription factors by deacetylation [26]. The upregulation of FOXO by SIRT1 enhances the cellular stress response, induces cell cycle arrest, resists oxidative stress, and assists in insulin signaling pathways to increase the expression of antioxidant enzymes [27]. These enzymes clear ROS and reduce oxidative stress-induced cellular damage, thereby delaying aging [28]. Furthermore, SIRT1 can augment the activation of NF-E2-related factor-2 (Nrf2) and its downstream key antioxidant gene, heme oxygenase-1 (HO-1), by deacetylating the transcription coactivator peroxisome proliferator-activated receptor gamma coactivator 1-alpha (PGC-1α). This regulation of the Nrf2 signaling pathway enhances cellular antioxidant capacity, protecting cells from oxidative damage [29].

Studies have shown that SIRT1 can also delay aging by regulating cellular autophagy. Autophagy is a crucial biological process where lysosomes digest and degrade cytoplasmic components, including organelles and soluble macromolecules. Autophagy-related genes (Atgs) and autophagy regulatory kinases are essential components of this process [30]. Organisms can clear damaged cytoplasm and protein aggregates within cells through autophagy. The overexpression or inhibition of autophagy in mammalian cells can promote or suppress the expression levels of aging-related proteins, thus playing a role in delaying aging. SIRT1 regulates autophagy through multiple mechanisms. For instance, it regulates AMPK, an important kinase that modulates energy homeostasis and is a key protein involved in various signaling pathways. It also plays a vital regulatory role in cellular senescence, and the activation of AMPK can delay or halt the aging process [31]. AMPK is the primary regulator of cellular energy metabolism and serves as a key sensor of the relative energy status upstream of mTOR in cells. In low-energy conditions, it activates autophagy with a high AMP/ATP ratio [32]. SIRT1 can activate AMPK, thereby inhibiting the mTOR signaling pathway and promoting autophagy [33,34]. Additionally, SIRT1 can enhance autophagy by deacetylating and regulating the activity of autophagy-related proteins. Autophagy is not only influenced by direct acetylation; the acetylation of certain transcription factors also plays a crucial role in this process [35]. On one hand, SIRT1 can directly affect autophagy by deacetylating key components of autophagy-related proteins, including the products of ATG5, ATG7, and ATG8 [36]. On the other hand, FOXO1 from the FOXO family is often reported to play a significant role in autophagy, with activated FOXO1 enhancing autophagy by increasing the expression of many autophagy-related genes in various cells [37]. Glucose deprivation (GD) increases FOXO1 deacetylation, and SIRT1 is essential for GD-induced FOXO1 deacetylation. Therefore, under GD conditions, SIRT1 can increase FOXO1 deacetylation, thereby increasing Rab7 expression. Rab7 is a small GTP-binding protein that mediates late-stage autophagosome–lysosome fusion, which is essential for the increased flux of FOXO1-induced autophagy [38].

In addition to regulating cellular metabolism, SIRT1 can also control the aging process by influencing cellular signaling pathways. For instance, SIRT1 deacetylates p53 (a tumor suppressor protein) in an NAD^+^-dependent manner, thereby inhibiting its transcriptional activity and pro-apoptotic function, which reduces cell apoptosis and maintains cell survival [39]. Moreover, a key reason for cellular aging is the accumulation of DNA damage. SIRT1 plays a crucial role in delaying cellular aging and preventing cancer by regulating DNA repair mechanisms, activating the DNA repair pathway, repairing damaged DNA, and maintaining genomic stability.

**Figure 1:**
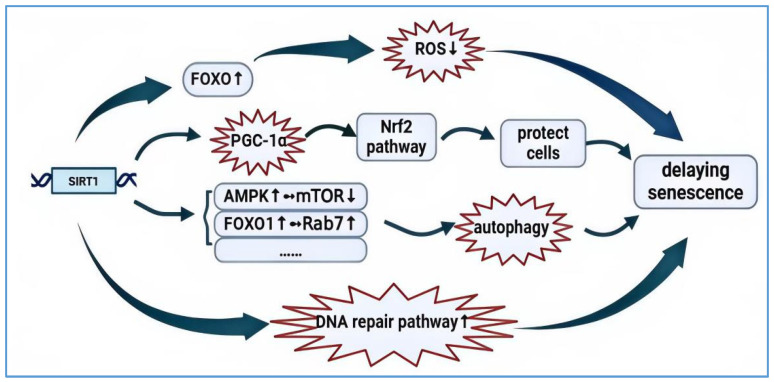
The mechanism by which aging is delayed by SIRT1. SIRT1, the most active regulator in aging research, possesses multiple anti-aging functions. Firstly, SIRT1 deficiency leads to increased oxidative stress levels, producing large amounts of ROS, which can cause cell and organ damage and accelerate the aging process. Upregulating SIRT1, on the other hand, enhances the FOXO family’s response to cellular stress, inducing cell cycle arrest and thus resisting oxidative stress and inhibiting excessive ROS production. Secondly, PGC-1α is a key target for delaying aging; SIRT1 can deacetylate PGC-1α, thereby regulating the Nrf2 pathway to protect cells. Additionally, autophagy, an important mechanism for clearing damaged cytoplasm and protein aggregates within cells, can either promote or inhibit the expression levels of aging-related proteins in mammalian cells through the overexpression or inhibition of autophagy, thus playing a role in delaying aging. SIRT1 can activate AMPK, among other mechanisms. Factors such as FOXO participate in the autophagy process. Finally, the accumulation of DNA damage is one of the key factors affecting cellular senescence. SIRT1 can regulate DNA repair mechanisms, activate the DNA repair pathway, and repair damaged DNA to maintain genomic stability, thereby contributing to the delay of cellular aging. SIRT1 plays a significant role in delaying organismal aging through these pathways. “↑” signifies activation; “↓” signifies inhibition; “……” signifies other pathways.

Beyond the research mentioned earlier, SIRT1 also plays a vital role in preventing stem cell aging and vascular aging. Firstly, SIRT1 functions as an anti-aging regulator for adult stem cells by modulating the gene activity that sustains stem cell function and delays cellular senescence through its regulatory effects on the AMPK and mTORC1 pathways, as well as by mediating gene expression [40]. Secondly, a decrease in SIRT1 expression levels with age can significantly impair vascular function; for instance, heightened endothelial dysfunction can contribute to the progression of cardiovascular diseases. It has been reported that SIRT1 can directly influence arterial endothelial function by deacetylating endothelial nitric oxide synthase (eNOS), which is activated to maintain vascular homeostasis through NO production [41]. The pathways through which SIRT1 contributes to delaying aging are illustrated in Figure 1.

### 2.3. The Relationship Between Mitochondria and Aging

Mitochondria, as important organelles within cells, are the core of cellular energy metabolism and play a crucial role in biological activities. They primarily produce ATP through oxidative phosphorylation, providing energy to the cell, and have earned the title of “the cell’s power plant” due to their critical role in cellular energy metabolism. As we age, the structure and function of our mitochondria undergo a series of changes, ultimately leading to age-related diseases such as diabetes, atherosclerosis, heart failure, and neurodegenerative disorders. Mitochondria not only play a vital role in energy production but also participate in various physiological processes, including cell signaling, apoptosis, and the regulation of oxidative stress. The loss of mitochondrial protein homeostasis, alterations in intercellular communication, the accumulation of mtDNA damage, and impaired mitochondrial biogenesis during aging can all lead to mitochondrial dysfunction, which in turn can cause senescence [5].

Mitochondria, known as the “powerhouses” within cells, primarily function through the tricarboxylic acid cycle (TCA cycle) and oxidative phosphorylation to produce adenosine triphosphate (ATP). These ATP molecules are essential for maintaining energy conversion and information exchange within cells, which are crucial for biological activities [42]. Research has indicated that in healthy cells, the transcription factor PGC-1α/NRF-1, encoded by nuclear genes, can regulate mitochondrial gene expression by inducing or increasing mitochondrial biogenesis and activity, thereby modulating cellular energy metabolism [43]. With advancing age, mitochondrial function gradually declines, leading to reduced ATP production, insufficient cellular energy supply, and decreased energy metabolism. This ultimately affects healthy cellular metabolism and repair, causing physical decline and accelerating the aging process.

Mitochondria produce reactive oxygen species (ROS) as a byproduct of energy metabolism, and these ROS can affect many critical intracellular components. For instance, they can directly impact the mitochondrial membrane and mtDNA near their site of production, leading to impaired mitochondrial function and increased ROS generation [44]. The primary role of ROS in the body is to act as signaling molecules or to introduce potentially harmful oxidative stress into cells. Low levels of ROS can enhance cellular defense mechanisms through the adaptive upregulation of antioxidant enzymes, thereby promoting health and longevity; however, excessively high levels of ROS can damage proteins, lipids, and DNA, causing significant oxidative stress and triggering the development of age-related diseases such as neurodegenerative disorders and cancer [45]. Under healthy conditions, ROS levels are low and can be cleared by the antioxidant system. Based on current evidence, increased mitochondrial ROS levels may not directly cause aging, but when combined with other factors, they do significantly contribute to the development of age-related diseases, such as epigenetic changes or weakened quality control systems [46].

Additionally, mitochondrial damage and dysfunction resulting from oxidative injury, membrane lipid peroxidation, a loss of the proton gradient, the depletion of cardiolipin, and mitochondrial DNA damage can be mitigated through the selective autophagy of mitochondria [47]. This process maintains mitochondrial quality control and homeostasis while regulating the production of new mitochondria, ensuring the health and vitality of mitochondrial function. Preclinical and clinical studies have indicated that impaired mitochondrial autophagy can negatively impact cellular health and may precipitate age-related chronic diseases [48]. The disruption of mitochondrial membrane potential is a critical factor in initiating mitochondrial autophagy [49]. The mitochondrial autophagy pathway can be categorized into PRKN (parkin RBR E3 ubiquitin ligase)-dependent and PRKN-independent mitochondrial autophagy. PRKN-dependent mitochondrial autophagy primarily involves the activation of PRKN, which recruits other autophagy-related proteins to the surface of damaged mitochondria, forming autophagosomes that subsequently fuse with lysosomes for mitochondrial degradation. The PINK1 (PTEN-induced putative kinase 1) and E3-ubiquitin ligase PRKN-mediated pathway is the most extensively researched in PRKN-dependent mitochondrial autophagy [50]. This pathway governs ubiquitin-dependent mitochondrial autophagy, affecting various physiological processes of mitochondria, including their biogenesis, dynamics, and autophagy mechanisms. The PRKN-independent mitochondrial autophagy pathway mainly depends on receptor proteins containing LIR motifs, which can directly interact with LC3 and GABARAP to facilitate the clearance of mitochondria [51]. Together, these two pathways are essential for maintaining mitochondrial health and function, which is vital for cellular health and the deceleration of aging processes. When the mitochondrial autophagy mechanism fails and cannot promptly eliminate damaged mitochondria, it can result in mitochondrial dysfunction and heightened oxidative stress levels, thereby accelerating the aging process and potentially triggering age-related diseases.

### 2.4. Interaction Between SIRT1 and Mitochondrial Biology

Mitochondrial biogenesis is the process by which cells generate new mitochondria to maintain homeostasis. This process relies on the timely and coordinated transcriptional control of both mitochondrial-encoded and nuclear-encoded genes [52]. The trigger mechanism is typically caused by increased cellular energy demand (such as from exercise or cold stimulation) or mitochondrial damage. Mitochondrial biogenesis encompasses the replication, transcription, and translation processes of mtDNA-encoded genes, the transport of phospholipids between organelles, and the protein translocation mechanism that transports nuclear-encoded proteins through the outer and inner membranes into mitochondria [53]. Peroxisome proliferator-activated receptor γ (PPAR-γ), a major regulator of mitochondrial biogenesis [54], enters the nucleus and binds to estrogen-related receptor-α (ERR-α), activating nuclear respiratory factors 1 (NRF-1) and 2 (NRF-2). This activation subsequently upregulates the expression of mitochondrial transcription factor A (TFAM), ultimately leading to the synthesis of mitochondrial DNA and proteins as well as the production of new mitochondria [55,56,57].

**Figure 2:**
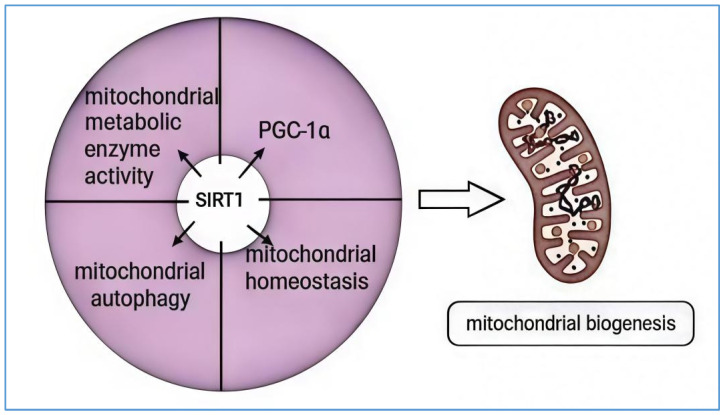
SIRT1 is implicated in the regulation of mitochondrial function and the main biogenesis pathways. SIRT1 plays a crucial role in mitochondrial biogenesis through the following mechanisms: First, mitochondrial fission and fusion, essential for maintaining mitochondrial function and balance, can be influenced by SIRT1 by regulating these processes. Second, SIRT1 regulates the activity and expression of mitochondrial autophagy-related proteins through deacetylation, thereby participating in the mitochondrial autophagy process. Third, SIRT1 activates metabolic enzymes and regulates their expression by deacetylation, contributing to mitochondrial biogenesis. Finally, SIRT1 deacetylates key regulators of mitochondrial biogenesis, such as PGC-1α, and modulates the expression of various genes involved in mitochondrial synthesis, thus promoting mitochondrial generation. SIRT1 primarily exerts its significant influence on mitochondrial biogenesis through these pathways.

SIRT1, an NAD^+^-dependent deacetylase, plays a crucial regulatory role in mitochondrial biogenesis. Initially, PGC-1α, a key regulator of mitochondrial biogenesis, undergoes various post-translational modifications, including acetylation and phosphorylation. Acetylation occurs on specific lysine residues and is catalyzed by histone deacetylases such as GCN5 [58]. This modification alters PGC-1α’s localization within the nucleus and inhibits its transcriptional activity. Conversely, the deacetylation of PGC-1α relies on the activity of SIRT1. SIRT1 facilitates the accumulation of PGC-1α in the nucleus, enhancing its deacetylation and increasing its activity, thereby promoting mitochondrial function and biogenesis [59]. For instance, in non-alcoholic fatty liver disease (NAFLD), activating SIRT1 can enhance the transcriptional regulation of genes involved in mitochondrial biogenesis, maintaining energy and metabolic stability. It also facilitates the deacetylation of downstream target proteins, such as PGC-1α, involved in fatty acid oxidation and mitochondrial function, which helps improve mitochondrial dysfunction [60]. Additionally, SIRT1 is important in regulating mitochondrial homeostasis, mitochondrial autophagy, and the activity of mitochondrial metabolic enzymes [61,62]. For example, nicotinamide mononucleotide (NMN) can activate the SIRT1-PGC-1α pathway, downregulate the expression of mitochondrial fission proteins (Drp1 and Fis1), and promote the expression of mitochondrial fusion proteins (Mfn1 and Mfn2). This action inhibits the mitochondrial dysfunction, apoptosis, and oxidative stress caused by pentylenetetrazol (PTZ) or free Mg2+ extracellular solutions [61]. Furthermore, SIRT1 has been shown to optimize mitochondrial oxidative metabolism and actively regulate autophagy and mitochondrial function under oxidative stress conditions. The overexpression of SIRT1 promotes the formation of autophagosomes and enhances the basal level of autophagy, while its deficiency hinders the response of autophagy to nutrient deprivation. In intestinal epithelial cells subjected to oxidative damage, SIRT1 may alleviate oxidative damage and maintain the integrity of intestinal epithelial cells by activating the autophagy/mitophagy mechanism [63]. Moreover, ROS, as byproducts of mitochondrial energy metabolism, have complex effects on mitochondria, involving both healthy physiological regulation and potential damage under pathological conditions. Superoxide dismutase 2 (SOD2), a key enzyme in regulating mROS production [64], can be deacetylated by SIRT1, which enhances the expression level of SOD proteins to inhibit lysosomal instability and mitochondrial damage caused by oxidative stress [65]. The primary pathways through which SIRT1 participates in mitochondrial biogenesis are illustrated in Figure 2.

## 3. The Aging of the Reproductive System

### 3.1. Organ System Aging and SIRT1

The aging of organs represents a systemic decline in bodily functions, and as time progresses, the risk of mortality or various diseases—including cancer, diabetes, cardiovascular diseases, or brain disorders—gradually increases [66]. The underlying cause is the accumulation of cellular damage and the diminishing capacity for regenerative repair. In vital organs such as the heart, liver, and kidneys, the number of functional cells irreversibly diminishes with age, and the fibrosis of the extracellular matrix accelerates, resulting in reduced organ elasticity and diminished metabolic efficiency. For instance, the apoptosis of cardiomyocytes weakens the heart’s pumping function, and the impaired regeneration of liver cells reduces their detoxification capacity. These organ-specific aging phenomena are closely associated with mechanisms such as the depletion of stem cell reserves and epigenetic dysregulation.

Environmental factors and endogenous stress collectively drive the aging process of organs. External factors, such as ultraviolet radiation and environmental pollution, trigger the mass production of reactive oxygen species (ROS), leading to oxidative damage to organelle structures and DNA double strands; internal factors include abnormalities in telomere dynamics and protein homeostasis, among other molecular-level disorders. Notably, different organs exhibit heterogeneity in their responses to damaging factors due to the non-renewable nature of neurons. Brain tissue is particularly sensitive to oxidative stress, whereas skeletal muscle is more susceptible to impaired mitochondrial autophagy function. This heterogeneity explains why the aging rates of different organs vary.

**Figure 3:**
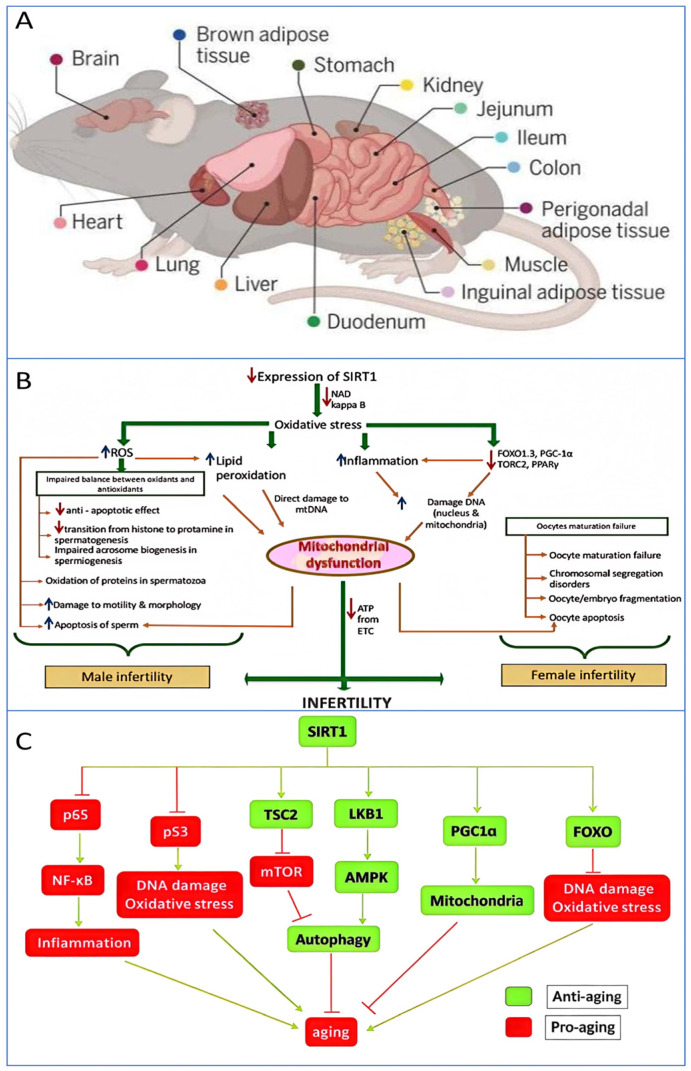
The cellular mechanisms of biological aging, the impact of reduced SIRT1 expression on the reproductive system, and the signaling pathways involving SIRT1 in aging. (**A**) This study analyzed 20 million cells from different life stages, genders, and genotypes across 623 mouse tissues. This massive dataset revealed over 3000 distinct cell states and more than 200 cell populations associated with aging. By clustering over 200 cell subtypes that experienced significant changes in aging-related groups, the nonlinear temporal dynamics of aging at the cellular level were uncovered. Additionally, by identifying over 200 different cell states with significant aging-related changes, this study aimed to discover key cell targets for therapeutic innovations aimed at restoring cellular function and biological processes in aging and disease [69]. (**B**) It illustrates that a decrease in SIRT1 expression results in an increase in reactive oxygen species (ROS) production, which causes lipid peroxidation and DNA damage in both male and female gametes (sperm and oocytes), thereby triggering infertility [70]. (**C**) This illustrates the mechanism of action of SIRT1 in the aging process of organisms. SIRT1 influences anti-aging activity by modulating various signaling pathways. In this Figure, arrows denote activation, and perpendicular lines indicate inhibition. The arrows indicate stimulation, and the lines with whiskers indicate inhibition [71].

Cell senescence, as a response to various stressors, can lead cells into a permanent state of proliferative arrest and is considered a key factor in aging and age-related diseases, making it an attractive target for therapeutic development [67]. Similarly, cell senescence is a critical mechanism for organ dysfunction, triggering a cascade of events through the secretion of senescence-associated secretory phenotypes (SASPs), which induces healthy cells to age [68]. When the cell cycle inhibitor p16INK4a and the p53/p21 pathway are continuously activated, senescent cells accumulate in large numbers within the organ microenvironment, not only losing their original functions but also releasing pro-inflammatory factors that damage surrounding cells. Recent studies have shown that the removal of senescent cells can significantly enhance the physiological functions of multiple organs in aged animals, offering theoretical support for intervention strategies targeting cell senescence, such as Senolytics drugs. These findings suggest that regulating cell senescence could be a key strategy for delaying organ degeneration. SIRT1, one of the most active proteins in aging research, is widely involved in cell proliferation, differentiation, apoptosis, and other biological processes, playing a crucial role in cell aging. Consequently, exploring the relationship between SIRT1 and aging, as well as cell aging, is of great significance for future anti-aging treatments. The cellular structure of biological senescence, the impact of reduced SIRT1 expression on the reproductive system, and the signaling pathways of SIRT1 involved in senescence are illustrated in Figure 3.

### 3.2. Aging of Female Reproductive Organs

The reproductive health and fertility of women are significantly affected by the aging of their reproductive organs. As age increases, phenomena such as the decline in ovarian reserve function, uterine atrophy, and the degeneration of fallopian tube function become gradually more apparent. In recent years, technological advancements in molecular biology and genomics have led to significant progress in research on the aging of female reproductive organs. Ovarian aging is considered the driving force behind female aging. The reproductive axis is one of the first organ systems to age. Reproductive aging is closely associated with the dysfunction of the hypothalamic–pituitary–ovarian axis, disruptions in the ovarian stroma, a decrease in both the quantity and quality of eggs, and changes in the morphology and function of the uterus. These factors collectively contribute to a weakened fertilization ability and impaired embryo development, ultimately affecting pregnancy outcomes and the health of offspring [72].

Recent studies have confirmed that the aging of female reproductive organs is closely related to biological processes such as oxidative stress, inflammatory response, telomere shortening, impaired autophagy, cellular aging, and mitochondrial dysfunction [73]. For instance, studies have shown that oxidative stress and inflammatory responses can stimulate the expression of the nerve growth factor-induced gene (Nur77) in the ovary. The overexpression of this gene can trigger mitochondrial autophagy, improve oxidative stress conditions, reduce apoptosis rate, and ultimately enhance the ovarian reserve in aged mice [74]. In addition, impaired autophagy plays a complex role in female pregnancy, ovarian function, gynecological malignancies, endometriosis, and infertility [75]. Within the ovaries, the quality of oocytes is a core factor determining female fertility and reproductive success [76]. Impaired autophagy can lead to the accumulation of damaged cellular structures (such as mitochondria) and harmful protein clumps, which in turn can cause oocyte aging and infertility [75]. With each occurrence of cell division, telomeres gradually shorten until they reach a critical length. At this point, cells lose their ability to divide, thereby triggering the aging of reproductive organs. The characteristics of cellular senescence are a decline in cell proliferation ability and abnormal functional manifestations. Meanwhile, mitochondrial dysfunction can lead to a disorder of energy metabolism, further affecting the healthy physiological functions of cells. These biological processes interact with each other and jointly promote the aging of female reproductive organs. Therefore, an in-depth exploration of the molecular mechanism of aging of female reproductive organs and a search for effective strategies to delay reproductive aging are of crucial significance for safeguarding women’s health and fertility.

### 3.3. Aging of Male Reproductive Organs

The aging of male reproductive organs refers to the gradual deterioration of their structure and function over time. This process not only impacts men’s fertility but may also lead to various reproductive system diseases. Although the universal mechanism of aging has been extensively studied, research on male reproductive aging remains inadequate. Currently, there is a growing awareness that the advanced age of fathers can have potentially serious implications for the health of their offspring, yet the understanding of how aging affects the male reproductive system is still limited [77]. Aging is a critical factor influencing male fertility, resulting in a decrease in both the quantity and quality of sperm, as well as a progressive decline in the structure and function of reproductive organs.

**Figure 4:**
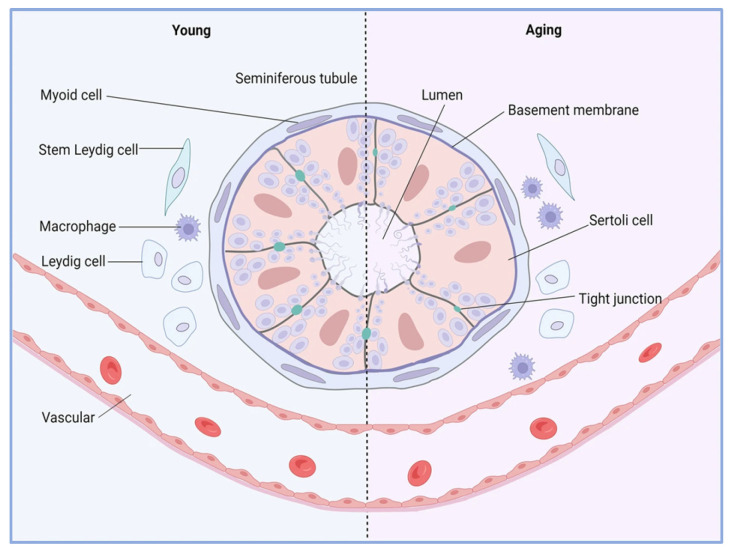
The dynamic interplay between homeostasis and aging in the testicular microenvironment. The testicular microenvironment comprises a precise regulatory system, which includes various cell populations with distinct functions and the active factors they secrete. With increasing age, a series of changes occur within the testicular microenvironment. A significant feature is the expansion of the pro-inflammatory-phenotype macrophage population, which triggers a cascade effect by disrupting the balance of the immune–reproductive axis’s homeostasis, adversely affecting other cell types should the supporting cells suffer dual damage (both in quantity and metabolism); if there is degeneration of the tight junction structure between cells; if the number and function of Leydig cells both decline; etc. [83].

As male reproductive organs age, the testicles, their primary reproductive glands, experience significant structural and functional changes that can affect fertility and overall reproductive health. Testicular aging is characterized by impaired spermatogenesis and decreased hormone secretion. A recent study revealed that men’s testicles undergo two major aging phases around the ages of 30 and 50. Notably, when the body mass index (BMI) surpasses 30, there is a significant reduction in both the quantity and quality of sperm [78]. Moreover, research has confirmed that the decline in mitochondrial function is a core factor driving testicular aging. For example, in a fruit fly model, the Vha68-3 gene is essential for maintaining mitochondrial derivatives. It facilitates the elongation of sperm cells during testicular aging by regulating mitochondrial metabolism, thus aiding in the preservation of male fertility [79]. The reduction in testosterone levels is also closely linked to male reproductive aging. As individuals age, serum testosterone levels tend to gradually diminish, and higher testosterone levels are associated with the retardation of biological aging. Hence, total testosterone has the potential to counteract the acceleration of biological aging [80]. During testicular aging, Leydig cells are particularly vulnerable to the aging process. In light of this, studies have found that the exogenous supplementation of β-hydroxybutyric acid (BHB) or the overexpression of the Hmgcs2 gene can decelerate the aging of Leydig cells and enhance testosterone production, thereby mitigating testicular aging [81]. Additionally, the targeted differentiation of testicular mesenchymal stem cells (such as intervention with melatonin drugs) can restore the differentiation capacity of these stem cells and also alleviate aging in the male reproductive system [82]. The age-related changes in the male testicular microenvironment are depicted in Figure 4.

## 4. Study on the Interaction Between SIRT1 and Mitochondria in the Aging of the Reproductive System

### 4.1. The Role of SIRT1 Interaction with Mitochondria in the Development of Germ Cells

#### 4.1.1. The Role of the SIRT1–Mitochondrial Axis in Oocyte Development

The senescence of oocytes is closely related to the decline in mitochondrial function. SIRT1, an NAD^+^-dependent deacetylase, can maintain the quality of oocytes by regulating mitochondrial autophagy and the stability of DNA. Studies have shown that SIRT1 plays a dual and spatiotemporal specific role in oocytes. Firstly, it can flexibly switch between epigenetic effects and non-epigenetic effects according to the process of meiosis [84]. In addition to regulating meiosis, SIRT1 also enhances the mitochondrial antioxidant capacity by increasing the activities of transcription factors FOXO3 and superoxide dismutase (SOD), protecting oocytes from oxidative stress damage [85]. Furthermore, SIRT1 can affect the expression of mitochondrial-related genes by regulating the three-dimensional structure of chromatin. For instance, the deletion of SIRT1 can lead to an increase in the acetylation levels of histones H3K9ac and H3K56ac, disrupting the higher structure of chromatin and thereby affecting the transcription of mitochondrial biosynthetic genes. Additionally, SIRT1 maintains the synergy between mitochondrial function and nuclear gene expression by regulating key signaling molecules of nuclear–mitochondrial communication (such as HIF-1), thereby influencing cellular senescence. Overall, in oocytes after ovulation, SIRT1 can delay cellular senescence by reducing the methylation levels of histones H3K9ac and H3K4, reducing ROS accumulation, improving spindle morphology, optimizing mitochondrial function, etc. [86]. Furthermore, an increasing number of current studies have shown that NAD^+^ deficiency is a common pathological factor in many diseases and aging processes [87]. As the coenzyme of SIRT1, changes in NAD^+^ levels directly affect the activity of SIRT1. In aged oocytes, a decrease in NAD^+^ levels can lead to a reduction in SIRT1 activity, causing the dysregulation of mitochondrial autophagy, resulting in abnormal mitochondrial accumulation and ROS accumulation, and ultimately leading to chromosomal abnormalities during meiosis and a decline in embryonic development potential. Evidence supports that knocking out the key enzymes involved in the new NAD^+^ synthesis pathway can lead to impaired SIRT1 function, which may trigger the phenotype of reproductive aging, resulting in a decline in the developmental potential of oocytes and impaired cell quality. The characteristics include spindle abnormalities and impaired mitochondrial function, ultimately reducing fertilization ability [88,89].

Granulosa cells within the follicular microenvironment can also influence the condition of oocytes. Research has indicated that the apoptosis of granulosa cells elevates the risk of oocyte apoptosis. The integrity of granulosa cells offers protection for oocytes in vitro, preventing oxidative damage. The production of ROS and the depletion of the antioxidant system contribute to the apoptosis of oocytes [90]. Moreover, the atypical apoptosis of granulosa cells can result in follicular failure, which can subsequently lead to ovarian aging and follicular atresia. By impeding the aging and apoptosis of granulosa cells, the progression of premature ovarian failure can be decelerated, as the regulatory mechanism of SIRT1/p53 is closely associated with the apoptosis of granulosa cells [91]. Specifically, when the expression of SIRT1 is increased, it facilitates the deacetylation of p53, thereby diminishing the transcriptional activity of p53 and its induction of downstream apoptosis-related genes, thus inhibiting the apoptosis of granulosa cells. Conversely, the opposite occurs. There is also evidence supporting that the biogenic pathway of mitochondria is a fundamental factor for follicular development [92]. Within mitochondria, PGC-1α and Nrf-1 can synergistically promote the expression of mitochondrial transcription factor A (TFAM). The activation of the TFAM promoter is essential for the transcription of both nuclear and mitochondrial genes and is a key mechanism of mitochondrial biogenesis. SIRT1 can directly interact with PGC-1α, resulting in the upregulation of PGC-1α expression and indirectly fostering mitochondrial biogenesis. However, the precise mechanism linking abnormal mitochondrial markers to the development of polycystic ovary syndrome (PCOS) is not yet fully understood [93]. Additionally, recent studies have discovered that SIRT1 can regulate various aspects of follicular development, encompassing the regulation of autophagy processes and the mitigation of oxidative stress. The restoration of autophagy function mediated by SIRT1 can suppress granulosa cell apoptosis induced by oxidative stress and assist in restoring their ability to secrete estradiol [94,95,96], ultimately decreasing the risk of oocyte apoptosis.

#### 4.1.2. The Role of the SIRT1–Mitochondrial Axis in Sperm Cell Development

While most research has concentrated on the female reproductive system, the role of SIRT1 in sperm cells is equally significant. Studies have confirmed that in mouse models, SIRT1 plays a crucial role in testicular development and spermatogenesis. It exhibits a high level of expression in spermatogonia and mature sperm, whereas its expression in spermatocytes and sperm cells is relatively low [97]. Immunohistochemical techniques have shown that SIRT1 is present in the nuclei of spermatogenic cells at various stages of spermatogenesis. The absence of SIRT1 results in the abnormal morphology of spermatocytes and increases the frequency of apoptosis in these cells [84,98]. Moreover, sperm motility is highly dependent on the ATP produced by the mitochondrial cristae structure, and the energy generated by mitochondria is utilized for the phosphorylation of flagellin [99]. SIRT1 may regulate mitochondrial biosynthesis by deacetylating PGC-1α, thereby maintaining the energy supply required for sperm flagellar movement. Additionally, the antioxidant function of SIRT1 can protect sperm mitochondria from DNA damage caused by reactive oxygen species (ROS), reducing the rate of sperm deformity. During the differentiation of spermatogonial stem cells, SIRT1 can influence chromatin remodeling by regulating histone acetylation modifications and may be implicated in the expression of meiosis-related genes.

### 4.2. SIRT1 Interacts with Mitochondria in the Aging Process of the Female Reproductive System

#### 4.2.1. SIRT1 Interacts with Mitochondria in Ovarian Aging

The ovary, as a crucial reproductive organ in females, bears significant responsibilities such as nurturing life and secreting hormones that maintain physiological balance. Its lifespan is a primary determinant of female reproductive function; ovarian aging is considered a continuous physiological phenomenon, with menopause marking the clinical end of ovarian function [100]. Evidence suggests that mitochondrial dysfunction promotes ovarian aging, with the main mechanisms driving this process including mitochondrial dysfunction, the accumulation of mtDNA mutations, impaired fusion and fission, metabolic changes, and defects in the electron transport chain (ETC), as well as reduced mtDNA levels [101]. The sirtuin family is considered one of the most important factors influencing both mitochondrial function and meiosis, and particularly SIRT1, which, besides regulating mitochondrial activity, is also regarded as one of the protective agents against oxidative stress in the ovaries, affecting oocyte age and progressive aging [102].

One of the key factors in the aging process is oxidative stress, and the loss of tissue and organ function is often caused by the excessive accumulation of ROS. This accumulation leads to oxidative damage, which in turn damages oocytes and granulosa cells in the ovaries, a critical factor for declining fertility [103]. In both biological and clinical studies, there is substantial evidence that increased levels of ROS can lead to follicular occlusion and ovarian oocyte aging. ROS, as inevitable byproducts of metabolism, are highly reactive oxygen-containing compounds, such as superoxide anions, hydrogen peroxide, and hydroxyl radicals, primarily produced continuously within cells through the electron transport chain (ETC) in mitochondria [104]. Under healthy conditions, cells can eliminate excess ROS, but when their production exceeds this level, they cause oxidative stress and cellular damage, leading to mitochondrial and nuclear DNA damage and cell apoptosis, and these types of damage have been shown to negatively impact the development and ovulation of ovarian follicles [105]. The SIRT1 pathway may exert potential protective effects on post-ovulatory oocyte aging by controlling ROS production [72], and evidence supports that inhibiting SIRT1 can suppress the oocyte upregulation of SOD2 and counteract the increase in ROS under oxidative stress conditions [106].

Additionally, maintaining mitochondrial homeostasis is essential for preserving the quality of oocytes in the ovaries and the developmental potential of embryos. Research has indicated that SIRT1 plays a pivotal role in the aging process of oocytes post-ovulation by regulating the distribution and function of mitochondria within the oocyte. In the case of unaged oocytes, mitochondria are evenly dispersed; however, in aged oocytes, they tend to aggregate into clusters, with a higher proportion of abnormal mitochondria compared to those in younger oocytes. Nevertheless, when SIRT1 is suppressed, the proportion of abnormal mitochondria significantly increases. Moreover, as oocytes age, the mitochondrial membrane potential declines substantially, but the activation of SIRT1 can restore this diminished membrane potential to healthy levels [107].

Mitochondrial dysfunction is a key driver of ovarian aging. However, selective mitochondrial autophagy can maintain the health of damaged mitochondria and preserve cellular vitality and function, playing a crucial role in delaying ovarian aging. Mitochondrial autophagy, a unique form of selective autophagy, primarily clears damaged or dysfunctional mitochondria. It involves the ubiquitin-dependent signaling pathway (PINK1-Parkin). When mitochondria are damaged, PINK1 accumulates on their depolarized outer membrane, recruiting and activating Parkin to ubiquitinate mitochondrial proteins. This ubiquitin signal marks the damaged mitochondria, allowing them to be recognized by autophagy receptors (such as p62/SQSTM1) and subsequently delivered to the autophagosome for degradation. Studies have shown that in aged mouse ovaries, the levels of PINK1 and Parkin decrease, leading to reduced mitochondrial autophagy activity, associated with ovarian aging [74]. SIRT1 can activate FOXO1 through deacetylation, thereby promoting the activation of the PINK1–Parkin pathway and enhancing mitochondrial autophagy. Additionally, SIRT1 can optimize mitochondrial oxidative metabolism and actively regulate autophagy and mitochondrial function under oxidative stress conditions; its overexpression promotes the formation of autophagosomes and increases the basal level of autophagy.

#### 4.2.2. SIRT1 Interacts with Mitochondria in the Aging of the Uterus

The uterus, an essential reproductive organ in females, primarily serves to provide an environment conducive to early embryo development, implantation, and the growth of the fetus and placenta. Uterine aging is a significant aspect of overall female aging, impacting fertility, and is closely associated with various gynecological diseases. The mechanisms behind uterine aging are influenced by numerous factors, and in comparison to ovarian aging, research on uterine aging has significant gaps. Evidence suggests that uterine aging is the underlying cause of numerous age-related pregnancy complications and congenital disabilities in offspring. The aging uterus primarily exhibits abnormal hormonal responses and diminished endometrial receptivity [108]. It is understood that mitochondrial dysfunction is a crucial factor in the aging process of multiple organs, yet the role of mitochondria in uterine aging is not fully comprehended. However, studies have indicated that mitochondrial dysfunction is closely correlated with ovarian aging, which in turn indirectly influences uterine function. SIRT1, a key longevity protein, also plays a critical role in uterine aging. Research indicates that SIRT1 is a pivotal driver of age-related PGR action necessary for the interaction between the uterine epithelium and stroma, and SIRT1 significantly influences age-related changes in the uterus, particularly for blastocyst implantation and stromal cell decidualization [109]. The overexpression of SIRT1 can prevent endometrial epithelial cells from aging, but the specific ablation of SIRT1 in the uterus accelerates premature aging in mice [109,110]. A uterine-specific p53 deficiency can also lead to uterine aging and premature birth in mice, while sirt1 can inhibit the p53-mediated senescence signaling pathway through deacetylation modification of the p53 protein.

Research into the impact of mitochondrial dysfunction and its interaction with SIRT1 on uterine aging has significant limitations, yet studies indicate that their interplay is crucial for understanding uterine aging. For instance, oxidative stress is a key factor in uterine aging. ROS, byproducts of mitochondrial metabolism, act as primary regulators for activating and modulating numerous signaling pathways. Elevated ROS levels can induce oxidative stress responses within cells, resulting in DNA damage, protein oxidation, and lipid peroxidation, which subsequently contribute to cellular aging and apoptosis. In the uterus, oxidative stress can alter structure and function, including endometrial shedding and implantation hinderance [111]. Oxidative damage is a major contributor to the loss of healthy function in the female reproductive system. SIRT1, an important antioxidant regulator, can enhance the activity of various antioxidant enzymes via deacetylation modifications, thereby mitigating oxidative stress-induced damage to the uterus. Despite the insights provided by research into the effects of mitochondrial dysfunction and SIRT1 interaction on uterine aging, many aspects remain enigmatic. For example, there is still a dearth in our detailed understanding regarding the specific mechanisms by which mitochondrial dysfunction influences uterine aging, especially its effects on energy metabolism and signal transduction in uterine cells. Furthermore, the regulatory mechanisms of SIRT1 in uterine aging are not fully elucidated, including its interactions with other signaling molecules and the specific targets of its action in uterine aging, which necessitate further research. Consequently, a thorough examination of the mechanisms involving mitochondrial dysfunction and SIRT1 interaction in uterine aging is anticipated to offer a novel perspective for the treatment of diseases associated with uterine aging.

### 4.3. SIRT1 Interacts with Mitochondria During the Aging Process of the Male Reproductive System

Currently, it is becoming increasingly common for fathers to have children at advanced ages, particularly in developed countries and regions. This trend has resulted in a rise in adverse reproductive outcomes, such as declining fertility rates, increasing miscarriage rates, and deteriorating child health conditions [112]. However, current analyses of the mechanisms involved in male reproductive system aging are still incomplete. The first evidence suggesting that sirtuins may play a role in male fertility control comes from mice carrying the SIRT1 allele [113]. Sirtuins are highly expressed in the testicular tissue of mammals, particularly in the nuclei of spermatogonia, spermatocytes, and round spermatids. This indicates that SIRT1 plays an intrinsic and direct role in the development of male germ cells during spermatogenesis [106].

Testicular aging is a significant manifestation of the decline in the male reproductive system, characterized by decreased sperm production and reduced sperm quality. Furthermore, the aging of the male reproductive system is closely associated with the decline in testosterone levels [82]. Testosterone, the primary male sex hormone, plays a crucial role in maintaining the development of sexual organs and healthy reproductive function [114]. As men age, the secretion of testosterone in their bodies gradually diminishes, which may affect sperm production and quality, leading to a decline in fertility. SIRT1, as a key regulatory factor, has garnered increasing attention for its role in the male reproductive system. SIRT1 may participate in the regulation of male reproductive system aging by modulating the synthesis and secretion of testosterone and influencing key signaling pathways during spermatogenesis. For instance, SIRT3, as part of the SIRT1 network that regulates oxidative stress and acts as an indicator of testicular metabolism, has its expression levels decrease, promoting glycolysis in rat testes [115]. In pre-diabetic rat models, PGC-1α, which activates Sirt3 transcription when SIRT1 is deacetylated, leads to a reduction in PGC-1α expression due to decreased SIRT1 activity, further causing a decrease in SIRT3 levels, thus exacerbating the negative impact of glucose metabolic defects on testicular mitochondria [116]. Additionally, studies have shown that the relative mRNA abundance of NRF-1 decreases in the testicular mitochondria of aged rats, and SIRT1 can stimulate the expression of NRF-1 and NRF-2 by converting inactive PGC-1α into its active form, which then acts on the nuclear gene [117] encoding the OXPHOS system, ultimately leading to the synthesis of mitochondrial DNA and protein, as well as the production of new mitochondria.

Mitophagy plays a crucial role in maintaining testicular cell homeostasis. Studies have shown that GCD contains components of lysosomes and mitochondria, with serine protease (HtrA2) being one of the main mitochondrial components. HtrA2 is highly enriched in the testes and exhibits genetic interactions with PINK1. PINK1 is a core element of the PINK1–Parkin signaling pathway, which is essential for mitochondrial quality control and autophagy processes. It functions not only within mitochondria but also plays a significant role in somatic cells; PINK1 kinase can phosphorylate Parkin on the outer mitochondrial membrane, activating E3 ubiquitin ligases to regulate mitochondrial protein degradation and promote mitochondrial autophagy [118]. SIRT1 can similarly activate this pathway to maintain mitochondrial health.

**Figure 5:**
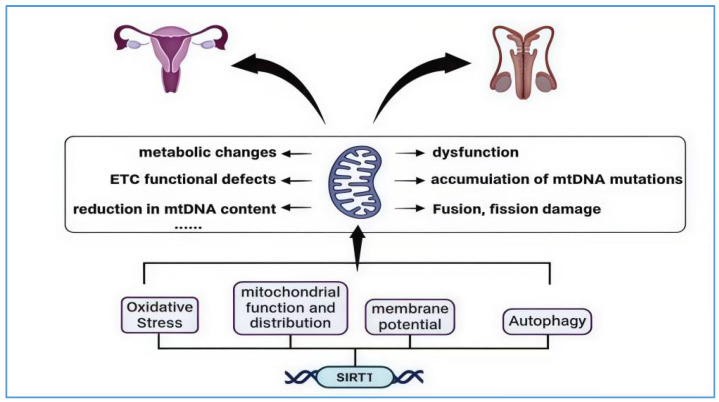
SIRT1 and mitochondria interaction in the aging of the reproductive system. The image illustrates the primary mechanisms through which mitochondria contribute to the aging of the reproductive system. These include mitochondrial dysfunction, the accumulation of mtDNA mutations, impaired fusion and fission processes, metabolic alterations, and defects in the electron transport chain (ETC), as well as diminished mtDNA levels. SIRT1’s interaction with mitochondria is primarily evident in its regulation of mitochondrial oxidative stress, mitochondrial function and distribution, changes in membrane potential, and autophagy processes. This interaction aims to reduce excessive ROS production, restore low membrane potential to healthy levels, and deacetylate and activate FOXO to promote the activation of the PINK1–Parkin pathway, thereby enhancing mitochondrial autophagy. The “……” in the figure represents other mechanisms by which mitochondria drive reproductive system aging.

During the aging process of the testes, the accumulation of ROS due to oxidative stress can severely damage germ cells. It has been reported that ROS are essential for spermatogonial cell differentiation and can disrupt the integrity of the blood–testis barrier, leading to reproductive toxicity [119]. Additionally, the accumulation of ROS-induced oxidative stress is the most prominent non-genetic factor in male infertility [120]. Under healthy physiological conditions, the small amount of ROS produced by sperm is necessary for fertilization, the acrosome reaction, and sperm capacitation [121]. However, if the production of ROS increases without a corresponding increase in the clearance rate of the removal system, it typically leads to peroxidative damage of the sperm plasma membrane and a loss of DNA integrity, resulting in cell death and reduced fertility [121]. SIRT1 enhances the expression of antioxidant enzymes by deacetylating the FOXO transcription factor, thereby more effectively responding to cellular stress, promoting cell cycle arrest, and thus resisting oxidative stress caused by ROS. The interaction between SIRT1 and mitochondria during reproductive system aging is depicted in Figure 5.

## 5. Conclusions

Aging, as a complex biological process, involves the interaction of multiple molecular mechanisms. Existing studies have shown that the interaction between SIRT1 and mitochondria plays an important role in aging. SIRT1 is mainly involved in the aging process of organisms by regulating mitochondrial biogenesis, mitochondrial homeostasis, mitochondrial autophagy, and oxidative stress responses, among other functions. Although it has been confirmed that the interaction between SIRT1 and mitochondria plays a role in the aging of various organs, the specific mechanism and role of SIRT1 in the aging of the reproductive system still need to be further explored, especially for males. This review aims to explore the impact of the interaction between SIRT1 and mitochondria on the reproductive system and reveal its key role in the aging process of the reproductive system. During the aging process of the reproductive system, the interaction mechanism between SIRT1 and mitochondria is particularly crucial. They jointly act on aspects such as energy metabolism, antioxidant defense, and the apoptosis of reproductive cells, thereby delaying the aging process of the reproductive system. Future research still needs to further explore in depth the specific molecular mechanisms of the interaction between SIRT1 and mitochondria, as well as the differences in these mechanisms among different genders and individuals and their application potential in anti-aging strategies, to provide more precise and effective guidance for clinical applications, reproductive health, etc.

## 6. Prospects

Mitochondria, known as “the powerhouses” of cells, are linked to the development of numerous diseases, including obesity, diabetes, and cancer. SIRT1, a type of deacetylase, plays a pivotal role in mitochondrial biogenesis. It has been identified as a potential therapeutic target for various diseases, such as chronic obstructive pulmonary disease and diabetic complications [122,123], and especially in age-related diseases. The mechanisms by which SIRT1 influences cell metabolism, aging, cardiovascular disease, inflammation, neurodegenerative disorders, and cancer have been extensively researched. Nonetheless, despite some progress in understanding how SIRT1 interacts with mitochondria to regulate reproductive system aging, significant gaps persist. In particular, the interaction between SIRT1 and mitochondrial biogenesis, as well as their impact on fertility, remains unclear in male reproductive systems. A deeper investigation into the role of SIRT1 in the aging process of the male reproductive system will not only help uncover the biological principles behind reproductive aging but also provide new insights for developing treatments targeting male reproductive aging-related diseases. Additionally, developing targeted therapies for SIRT1, such as pharmacological interventions and stem cell therapy, to slow down the aging of the reproductive system and enhance fertility will be an important area of research, offering new strategies for treating age-related reproductive diseases.
